# Gentrification and Chronic Conditions in Older Adults: Service Providers’ Perspectives

**DOI:** 10.1093/geroni/igaa057.1095

**Published:** 2020-12-16

**Authors:** Ronica Rooks

**Affiliations:** University of Colorado Denver, Denver, Colorado, United States

## Abstract

Where we live impacts our health, but this is more apt for older adults (aged 55+) aging-in-place in their neighborhoods. Gentrification, i.e. the transformation of neighborhoods from low to high value, can put community-dwelling older adults at risk for residential displacement with limited retirement incomes and financial stressors like increased housing costs and property taxes, residential turnover and changing access to resources. As a place-based stressor, gentrification may exacerbate social vulnerabilities (e.g., lower socioeconomic status and racial/ethnic minority status) related to chronic condition (CC) disparities. But, little gentrification research focuses on these issues. This research examines associations between gentrification and older adults’ CC management related to broader social determinants in Hamilton, Ontario, Canada from health and social service providers’ perspectives. Hamilton, a recovering steel industry city with in-migration from Toronto, is experiencing higher costs of living, income inequality and tension with recent gentrifiers. I conducted key informant interviews with service providers in city government and community-based organizations using thematic analysis. Across providers, food insecurity, social isolation and displacement were the biggest issues associated with gentrification and CC, particularly for older adults with lower incomes and government disability support. Results thus far reveal Hamilton has numerous older adult-focused providers, but older adults often have difficulties accessing services due to a lack of knowledge, not always asking or realizing when they need help and coordinated referral difficulties across providers. To address these challenges, providers consider environmental scans, mapping resources and advertisement in an online community information database from the city’s public library.

